# *Paniya Voices*: A Participatory Poverty and Health Assessment among a marginalized South Indian tribal population

**DOI:** 10.1186/1471-2458-10-149

**Published:** 2010-03-22

**Authors:** KS Mohindra, D Narayana, CK Harikrishnadas, SS Anushreedha, Slim Haddad

**Affiliations:** 1 Centre de recherche du Centre hospitalier de l'Université de Montréal, Montréal, Canada; 2 Institute of Population Health, University of Ottawa, Ottawa, Canada; 3 Centre for Development Studies, Thiruvananthapuram, India; 4 Centre for Development Studies local office, Wayanad, India; 5 Groupe de recherche interdisciplinaire en santé, Université de Montréal, Montréal, Canada

## Abstract

**Background:**

In India, indigenous populations, known as *Adivasi *or Scheduled Tribes (STs), are among the poorest and most marginalized groups. 'Deprived' ST groups tend to display high levels of resignation and to lack the capacity to aspire; consequently their health perceptions often do not adequately correspond to their real health needs. Moreover, similar to indigenous populations elsewhere, STs often have little opportunity to voice perspectives framed within their own cultural worldviews. We undertook a study to gather policy-relevant data on the views, experiences, and priorities of a marginalized and previously enslaved tribal group in South India, the Paniyas, who have little 'voice' or power over their own situation.

**Methods/design:**

We implemented a Participatory Poverty and Health Assessment (PPHA). We adopted guiding principles and an ethical code that promote respect for Paniya culture and values. The PPHA, informed by a vulnerability framework, addressed five key themes (health and illness, well-being, institutions, education, gender) using participatory approaches and qualitative methods. We implemented the PPHA in five Paniya colonies (clusters of houses in a small geographical area) in a *gram panchayat *(lowest level decentralized territorial unit) to generate data that can be quickly disseminated to decision-makers through interactive workshops and public forums.

**Preliminary findings:**

Findings indicated that the Paniyas are caught in multiple 'vulnerability traps', that is, they view their situation as vicious cycles from which it is difficult to break free.

**Conclusion:**

The PPHA is a potentially useful approach for global health researchers working with marginalized communities to implement research initiatives that will address those communities' health needs in an ethical and culturally appropriate manner.

## Background

A key public health challenge is to determine the health needs of indigenous populations using approaches that appropriately reflect their conditions and concerns while respecting their culture and identity. Indigenous researchers have challenged approaches that are rooted in scientific objectivity, calling for the use of indigenous theoretical frameworks, perspectives, and 'ways of knowing' [1,2]. Furthermore, they argue that research initiatives should focus on indigenous research priorities that promote self-determination, mobilization and transformation. Previous health research with indigenous populations has been criticized for lacking cultural sensitivity, inadequately addressing indigenous views of health and illness, using unethical practices, not sufficiently engaging the participation of indigenous communities, and having limited impact on policy and action, among others [3-6]. While researchers in wealthier countries have begun to address these critiques, [7-9] approaches still need to be developed for conducting research among indigenous populations in low-income countries, where resources for conducting research are particularly limited. This paper presents an approach we developed in rural India to address this gap.

In India, indigenous populations, known as *Adivasi *or Scheduled Tribes (STs), are among the poorest and most marginalized groups [10]. National level data show that STs have higher mortality rates than non-STs, even after adjusting for living standards [11]. Epidemiological studies have concluded that ST populations face more risks of ill health compared to other social groups; among other things, they are more likely to smoke and consume alcohol, [12,13] and they have higher rates of morbidity [14]. Despite more than 50 years of affirmative action by the Indian government, large disparities in health and well-being persist between STs and the rest of the population [10].

STs and their indigenous perspectives have long been marginalized; assessing their health needs therefore requires an appropriate, culturally sensitive methodology. Moreover, 'deprived' (i.e., historically oppressed and extremely poor) ST groups tend to display high levels of resignation and to lack the capacity to aspire; consequently, their health perceptions do not adequately represent their real health needs [14,15]. Finally, like indigenous populations elsewhere in the world [3], STs have little opportunity to voice perspectives framed within their cultural worldviews.

Researchers are increasingly advocating participatory approaches with indigenous populations, that respect their self-determination and the right to control their own research and policy priorities [16]. We developed an approach, the Participatory Poverty and Health Assessment (PPHA), that draws upon public health traditions and development research and practices. The PPHA is rooted in the philosophy that those most affected by health and development issues should be active participants in the research process and subsequent policy action [17-19]. The PPHA was inspired by the World Bank's project *Voices of the Poor*, which investigated the realities of poor people in 50 countries using participatory and qualitative methods [20,21].

The PPHA encompasses approaches commonly used in participatory rural appraisal (PRA), which involves local people in assessing their own situation [18]. However, unlike PRA, which is generally insufficiently theorized [22], the PPHA is theoretically grounded. In the PPHA, marginalized groups participate in the analysis of their own situation by exploring local meanings of health and poverty and the connections between health and poverty. Standardized questionnaires present challenges related to face validity, which the PPHA approach circumvents by putting indigenous perceptions into context. This is especially relevant when studying populations with divergent worldviews. The aim of the PPHA is to collect in-depth information that can be quickly disseminated to decision-makers.

This paper describes how we used PPHA with a marginalized tribal group to gather information on their views, experiences and priorities that can help in developing more appropriate interventions and policies.

## Methods

### Setting

The current study, *Paniya Voices*, is part of an action research project, *Vulnerability and Health in Wayanad, Kerala*, implemented by the Centre for Development Studies and the Université de Montréal. Wayanad, the mountainous northern district of Kerala, has an agricultural economy and is home to about one-third of the state's ST population. The low literacy among the STs contributes to Wayanad's having relatively lower literacy rates than Kerala's other districts. Among the population actively engaged in the work force, agriculture is the main occupation. The study site is Kottathara, a *gram panchayat *(the lowest territorial decentralized unit) with a land area of 31.75 km^2 ^and a population of about 17,000. We have been working with the community in Kottathara since 2002 to improve evidence-based policy-making and to devise and implement a community-based health insurance (CBHI) scheme to reduce inequities in access to health care. The CBHI (known as SNEHA) has been implemented and is now an independent body, run for and by community members. Members of the Paniya tribe, however, have not participated in this organization, despite their high levels of poverty and health needs. Further, our initial study of the Paniyas' health status, using conventional surveys with standardized questions that are typically used at the population level to assess self-reported health, suggested their status to be better than what was assessed clinically. Clearly, to understand their situation we needed a different approach.

### Study population

The Paniya tribe was previously enslaved by upper castes, is extremely marginalized and deprived [23]. The Paniyas live in colonies (clusters of houses in a small geographical area) in peripheral areas; they rarely interact socially outside their own colony. Colonies have poor transportation linkages and are particularly vulnerable to flooding during monsoons. Paniyas are predominantly landless--75% of Paniya households each own less than 10 *cents *of land (100 *cents *being an acre)--and have poor housing and living conditions; for example, 50% of households have no sanitation facilities [24]. Paniyas have low levels of education; 57% of women and 46% of men have never been to school. They spend a significant proportion of their household income on alcohol and tobacco, which represent 17% of total expenditure on food consumption. Hygienic practices common in Kerala are not universally adopted; over a quarter of the households do not systematically boil their drinking water. Their health needs are great (e.g. 60% are underweight, 15% are anaemic, 11% have a goitre) [25,26]. The Paniyas have low rates of health care utilization; among those who had experienced a severe episode of illness, 30% did not use any health service [27]. Although there are special tribal schemes and programs, the Paniyas are less likely to avail themselves of these, compared to other tribal groups.

Finally, the Paniyas also demonstrate high levels of resignation to their situation and have been found to underreport their health conditions, which is an indication of their extreme levels of deprivation and marginalization [28].

### Guiding principles in PPHA implementation

Researchers undertaking participatory research with indigenous populations have argued for explicit principles to guide such participatory initiatives. For example, in Canada, the Kahnawake Schools Diabetes Prevention Project identified four guiding principles, which included integrating participants as equal partners and creating new learning opportunities for them [8].

We adopted three principles that we have found useful for promoting Paniya participation and autonomy: respect for Paniya culture, relationships of trust, and flexibility. These principles, which are important elements of social justice [29], improved the effectiveness of the study's implementation. We developed these principles in conjunction with a local non-governmental organization (NGO) that undertook the field work. The NGO's field team consisted of two men from the study area and two women from other districts in Kerala who lived in the study area for the duration of the field work (eight months).

First, recognizing that the Paniyas have historically been marginalized and oppressed, we have operated under an explicit principle of respect for their culture. Thus, our first interactions with the Paniya colonies were with their traditional leaders (*moopans*). We also strove to preserve the meanings of the terms used by Paniyas and to ensure their voices would be truly heard, by working exclusively with this particular tribal group and by engaging a field team that could work in their language. The Paniyas speak a distinct language that is not well understood and sometimes ridiculed by non-Paniyas. Two members of the field team were fluent in this language and able to communicate directly in it. Data were recorded in the Paniya language and the data analyses integrated precise Paniya sayings.

Second, because of their history of oppression and persistent experience of discrimination, the Paniyas greatly distrust the outside community. Therefore, we made trust-building a cornerstone of our initiative. Prior to any data collection, the field team met several times with Paniya leaders and had informal discussions with colony members (5 to 10 informal visits to each colony). The field team undertook these interactions with the aid of ST promoters--individuals hired by the *gram panchayat *to interface with the ST colonies and to enhance the effectiveness of ST development schemes--and of other community members trusted by the Paniyas. We also adopted ethical procedures (described below) that helped to build trust with the Paniya community.

Third, like other researchers, [8] we found *flexibility *to be essential in implementing the study. Having done all the careful planning required for successful interventions, we needed to diverge somewhat from our original plan to adapt to contextual factors. For example, we had intended to carry out the PPHA on a cross-colony basis. However, from preliminary attempts to conduct cross-colony meetings, we understood this was clearly not feasible because of large distances and poor transportation between colonies, lengthy work days (Paniyas being predominantly wage labourers), and the Paniyas' fear of leaving their colonies. Therefore, we conducted the PPHAs at the individual colony level. This approach had its drawbacks, as the participants knew each other well and group activities (see section on data collection) were often heterogeneous in terms of gender and age. However, this approach opened up new opportunities. Carrying out colony-level PPHAs enabled us to build trust within the colonies, gather in-depth information on each colony selected, and increase the validity of our findings by regularly cross-checking information during weekly colony visits. Over time, the Paniyas became more comfortable with the field team and the activities, which made it possible to pursue several cross-colony activities, such as group ranking exercises.

### Ethics

Recognizing the Paniyas' vulnerability and previous negative experiences with persons from outside the community, as well as the collective nature of their society, we formulated our ethical procedures in two stages. First, we developed and implemented a code of research ethics with guiding principles and practices to ensure the research partners achieve the study's objectives respectfully and ethically. Our ethical code has two main parts. The first sets out 13 guiding principles for working with the Paniya communities in the study. These principles relate to confidentiality, voluntariness of participation, and informed consent, as well as to respect for Paniya culture and empowerment dimensions. The second part of the code details the obligations of each of the partners in the study--not only the researchers and the participating Paniya colonies, but also the NGO and its field research team. Because of their history of oppressive relations, we paid particular attention to ensuring Paniyas could control the nature of their interactions. For example, the field team was obliged to leave the colony at any time if the Paniyas felt exposed to unfavourable attitudes or practices. This code is a written agreement that is flexible and meant to be revisited with the participants throughout the study. We developed it using an interactive process: it was first drafted by the researchers and then revised by the NGO. Future research initiatives could involve the participants of the study in drafting of the code if sufficient time is allotted to this activity prior to the study.

Once the ethical code had been developed, we presented it to the Paniyas as part of the process of collecting community consent--the second stage of our ethics procedures. Like other indigenous groups, the Paniyas tend to organize social norms and values around collective principles governing such things as living arrangements and decision-making procedures. Therefore, we sought community consent for the study from each colony prior to soliciting individual consent, so that the Paniyas could decide whether this study was in the best interest of their community. Colonies gave their consent based on discussions at the colony level and the support of community representatives. For community consent, we obtained the *moopan*'s signature at colony-level meetings. We based our process on experiences and guidelines developed in the Canadian context with indigenous populations [7,30], which we adapted to the local context, taking into account factors such as the Paniyas' low literacy levels. We completed the ethics procedures by the first week of April 2008, prior to beginning official data collection.

The *Paniya Voices *study was approved by the ethics committee of the Research Centre of the *Centre hospitalier de l'Université de Montreal*.

### Sampling of participating colonies

**Figure 1:**
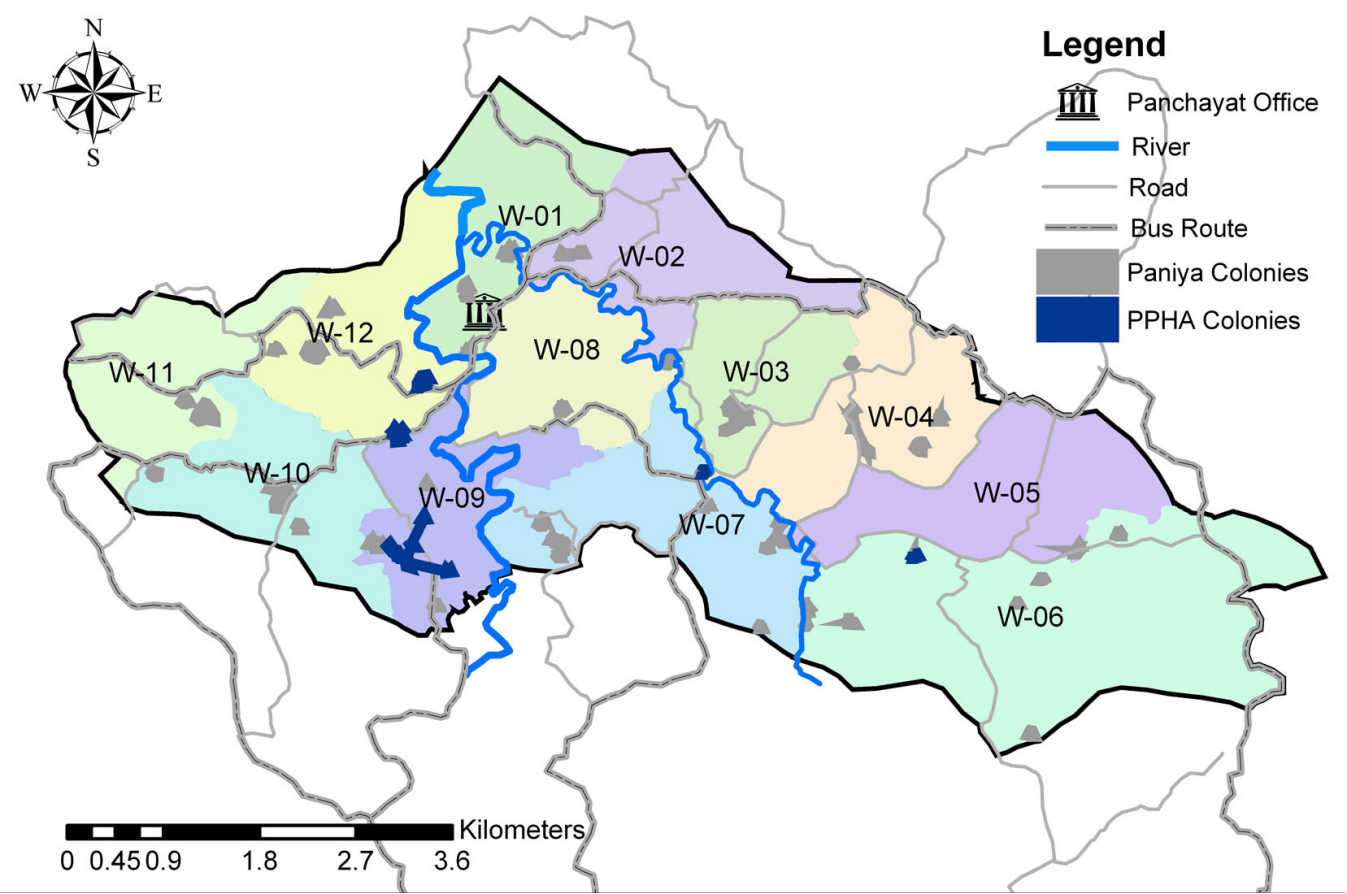
**Map of Paniya colonies and participating *Paniya Voices *colonies, Kottathara Panchayat, India**.

We used purposive sampling to select colonies. Our aim was to diversify our sample in terms of colony size, distance to basic services, and living standards. Of the 45 colonies identified in the *gram panchayat*, we selected five in which to collect in-depth information for the study (Figure 1) [31]. Every household in each colony was invited to participate in the PPHA. All adult (> 15 years) household members could participate in the activities, although children were often present. We undertook the PPHA activities over several months to accommodate the Paniyas' working schedules. Respondents for individual interviews were selected based on their life situations, which became known to the field team over the course of their interactions with the colonies. Participants in group interviews were typically convenience samples of colony members available on any given day.

### Description of the participating colonies

Here we briefly describe each colony, using pseudonyms to protect anonymity. Alathottam (five households) reports no safe water throughout the year and no electricity supply; houses are currently being electrified. Olamala (26 households) sits on top of a hill and is spread out; the lack of nearby water precludes the use of latrines. Cheruvayal (six households) sits below road level and is prone to flooding. Only two of its houses are electrified, and although there is a well nearby, the water is not good. The colony's major problem is the nearby liquor shop. Unlike the others, Puthiyamala (12 households) is a mixed colony, with five households having Scheduled Caste affiliations. It is electrified, and all the households have sanitation facilities and good water supply. Vayal (17 households) is close to various services, such as the primary health centre (PHC), *anganwadis *(preschools) and the *panchayat *office, but they still lack basic facilities, including electricity and latrines. This colony is prone to flooding during monsoon season, resulting in isolation and a recurrent need to rebuild after each flood.

### Data collection

We conducted a feasibility study, consisting of a full day event, on April 26, 2006, with 14 *moopans *and other senior male Paniyas. The participants were mobilized by an educated Paniya man from Kottathara who had previously worked as an ST promoter. He coordinated and facilitated the event, along with a graduate student with ST affiliations from another district. This feasibility study had two parts. First we explained the study's objectives and format, and affirmed our intention to work with the Paniya colonies to produce change and not merely to undertake a research study. Then we conducted a number of group-level PPHA activities, including mapping, ranking exercises, and group discussions. We found that these activities rapidly elicited good quality data. Furthermore, this study both confirmed the Paniya community's interest in participating in *Paniya Voices *and rallied the traditional leaders' support.

The PPHA field work was undertaken by our NGO partner, who has expertise in participatory methods and experience in working with tribal populations. We conducted additional training sessions with the field team in detailed note-taking, transcribing, recording field notes, translation procedures, and data storage, to ensure field activities followed proper research standards, which differed from their standard NGO procedures, such as maintaining data confidentiality and producing exact verbatim transcriptions rather than summaries of interviews and group discussions.

The PPHA investigated the underlying processes of poverty, ill health, and vulnerability. The investigation was guided by a vulnerability framework based upon multiple disciplines, including public health, biomedicine, economics, and environmental science [32]. Vulnerability refers to two convergent processes: an elevated exposure to a risk and an incapacity to reduce the potential deleterious consequences of exposure to that risk. A person or group exposed to risk factors without access to resources and basic services such as quality health care can be considered vulnerable. We explored how the Paniyas viewed their situation with respect to these two processes; our ultimate goal was to identify potential interventions that would help to overcome this vulnerability.

We selected specific themes to explore based on previous survey findings, the feasibility study, and preliminary field work. We investigated the following key themes:

- *Health and illness*: how Paniyas experience ill health, based on local understandings of health problems, causes and consequences; season-related issues; coping strategies employed when a household member falls ill; access to health care and treatment-seeking behaviour.

- *Well-being*: how well-being is defined and categorized by Paniyas; identification of resources in colonies; the relationship between Paniyas and these resources; main challenges to living a good life; changes over time and across seasons; hopes for the future.

- *Institutions*: which public institutions play an important role in the Paniyas' lives, and how the Paniyas rank them in terms of effective service provision.

- *Education*: reasons for the high drop-out rates of Paniya children; barriers preventing them from obtaining an education; relationships between teachers and the Paniya community.

- *Gender*: while gender was not a specific theme, we explored it as a cross-cutting issue across the four main themes seen in differences between men and women, and boys and girls, as well as in intra-household dynamics and issues such as male alcohol use.

**Figure 2:**
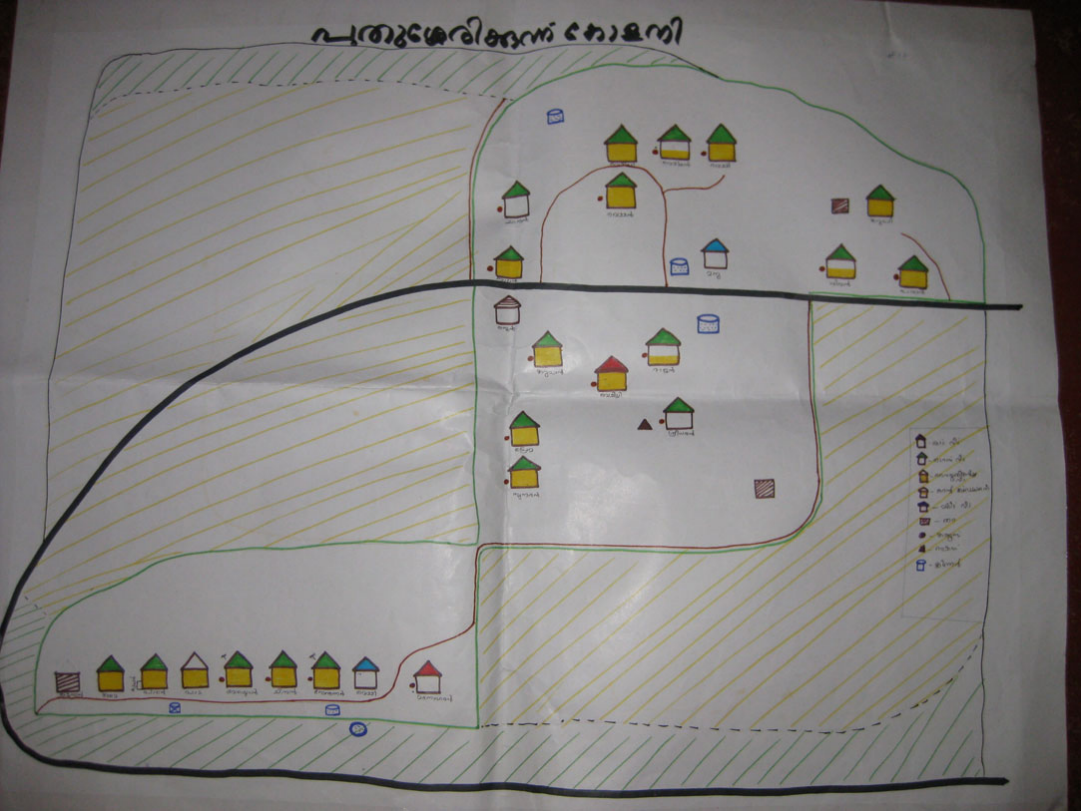
**Example of a social resource map as part of *Paniya Voices*, Kottathara Panchayat, India**.

**Figure 3:**
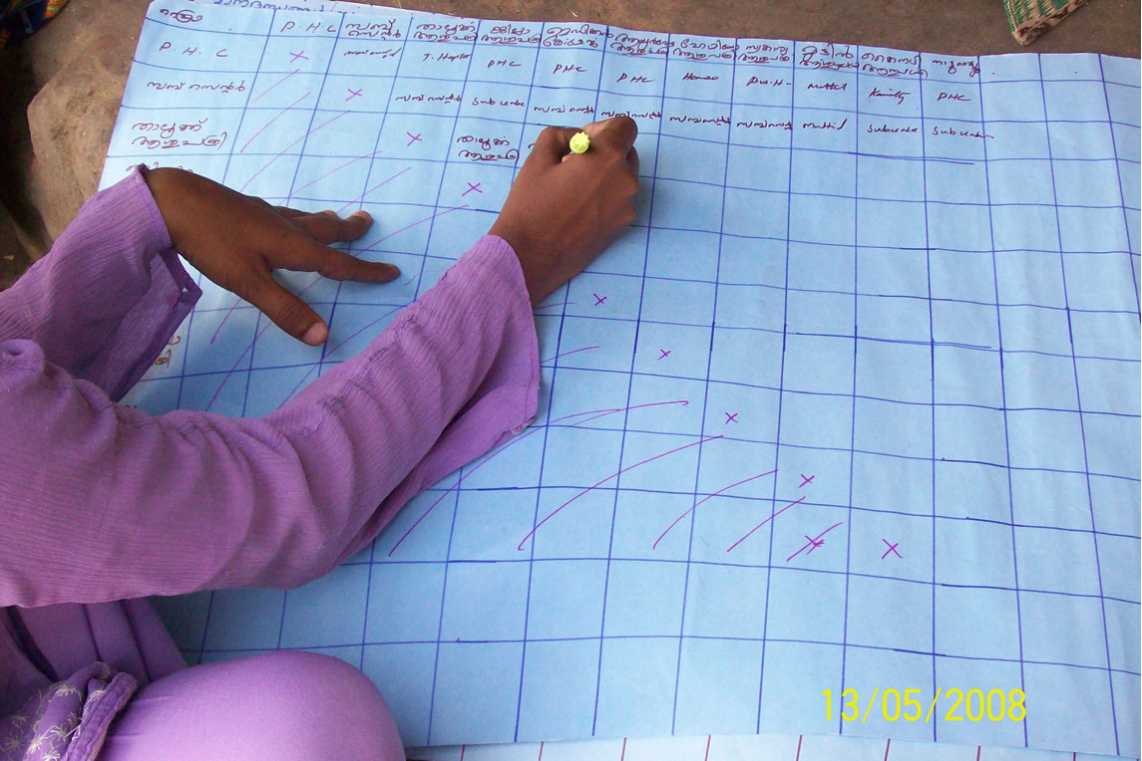
**Example of a relationship mapping exercise as part of *Paniya Voices*, Kottathara Panchayat, India**.

The field team used the PPHA to explore these themes through a combination of participatory approaches and qualitative methods (focus group discussions, semi-structured interviews). The participatory approaches are detailed in Table 1. These activities aim to spur participants to conceptualize and assess their situation along several dimensions, including space (mapping of resources, mapping of mobility of individuals, transect walks), time (tracing events such as illness or food availability by season, daily routine analysis that tracks time spent on specific activities), and relationship (matrixes that rank preferences or priorities, cause-effect and impact analyses to distinguish between causes of problems). Figure 2 presents an example of a spatial activity. Participants mapped their respective colonies, including risk items such as sources of flooding during monsoon or a nearby liquor shop. Figure 3 presents an example of a relationship activity. Participants ranked the various health institutions according to their preferences for access and quality. Certain approaches yield similar information, which can be used to cross-check findings over the study period.

**Table 1:** Participatory approaches for PPHA

Approach	Description	Themes addressed
Transect walk	Walk through colony to meet informally with colony members and observe infrastructure and basic amenities (e.g. electricity, water source).	General information
Local history	Informal discussions with senior colony members on the colony, settlement patterns, housing patterns, etc.	General information
Mapping of social resources	Map key buildings, infrastructure, basic amenities, housing and land patterns, etc., within their colony.	General information, Well-being
Coverage and service matrixes	Identify key institutions, distance to institutions, travel costs, type of services available, perceived quality.	Institutions, Health, Education
Matrix ranking	Comparative ranking and prioritization of quality of different service providers and facilities in the institutions.	Institutions, Health, Education
Mobility mapping	Mapping of distances to institutions based on different needs and identification of barriers in travelling to institutions.	Institutions, Health, Education, Well-being
Force field analysis	Tracing positive and negative factors and forces in place (in and outside the community) regarding situation.	Health, Education, Well-being
Seasonality analysis	Tracing patterns of outcomes (e.g. food security) over the period of one year across seasons. Examine effects for different groups (e.g. men, women, children).	Health, Education, Well-being
Cause effect and impact analysis	Trace causes of key problems (e.g. illness) and explore impacts of problems on different aspects of life.	Health, Education, Well-being
Time line and trend analysis	Trace changes in outcomes (e.g. food security) and beliefs (e.g. spiritual) over different periods of time (e.g. past 30 years).	Health, Education, Well-being
Wealth ranking	Identify different well-being categories with criteria of individuals or households based on participants' own categories, then count the number in each category.	Well-being
Daily routine analysis	Outline daily activities during a period of 24 hours for individual men and women. Assess characteristics of activities (e.g. high energy tasks, poverty reduction, caring, etc).	Well-being
Power relation matrix	Identify institutions over which participants feel they have control and those over which they would like to have more.	Institutions

### Data recording, management and analysis

The field team recorded data and field notes in handwritten diaries. They translated transcripts into English; the researchers reviewed these transcriptions to ensure a logical translation and back-translated selected transcripts to validate accuracy. We set up a data management system to be able to store data confidentially and retrieve it easily for analysis. The field team clarified any uncertainties during return trips to the colonies. They also consulted ST promoters periodically to ensure we had a proper cultural understanding of the data.

Two of the authors performed data analysis--in this case a thematic framework analysis [33] guided by the vulnerability framework. Analysis involved iterative processes between coding (using a structured codebook), thematic framework, descriptive accounts, and interpretative analysis. Following this analysis, the research team met with each of the participating colonies to present and discuss our interpretation of the findings. We also presented the findings at a workshop to Paniya representatives from other localities and local NGOs who have long experience working with Paniya communities. In these discussions, our aims were to increase the validity of the findings and to promote their appropriation by the Paniyas. In a next stage of analysis, we will merge the findings with quantitative data (including baseline, panel and clinical health surveys) gathered by the project, in order to provide clearer policy advice [34].

### Preliminary findings

**Figure 4:**
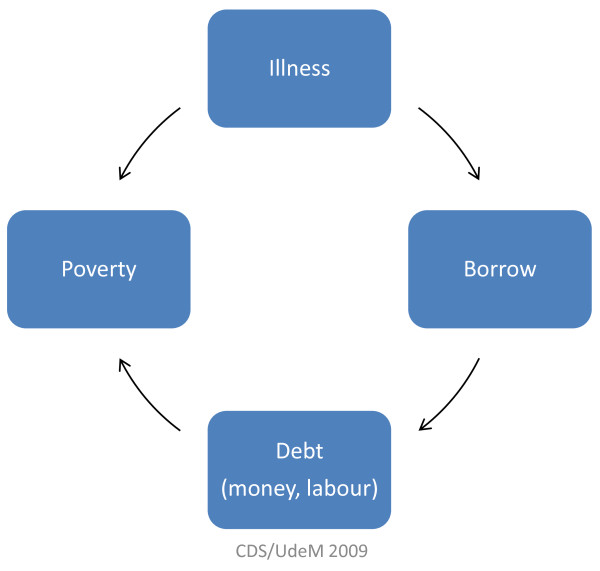
**Example of a 'vulnerability trap' as part of *Paniya Voices*, Kottathara Panchayat, India**.

This analysis uncovered that the Paniyas view their situation as vicious cycles from which it is difficult to break free. We identified a number of different 'vulnerability traps' and summarized them in simple diagrams to facilitate presentation to the Paniya participants and the larger Paniya community. An example of a trap is presented in Figure 4. Illness is a major source of vulnerability that leads to poverty by two different paths. The first is direct, which is the incapacity to work. The second, indirect path is indebtedness incurred by borrowing to pay for health care costs. More in-depth analysis of this trap revealed that the Paniyas overwhelmingly rely on borrowing from outside their community, typically from nearby land-owners or from their employers. Consequently they become indebted in terms of labour owed, reproducing historical oppressive relationships [35]. The Paniyas identified several other vulnerability traps related to a range of risk factors, including landlessness, poverty, exposure to harsh environmental conditions (e.g. floods), alcohol use, colony isolation, and education deficits. Once they have fallen into such traps, it becomes increasingly difficult to protect themselves from the various risk factors, thereby further reducing levels of well-being and paving the way for the 'road to destitution'[36]. These findings suggest that reducing the Paniyas' vulnerability will require breaking these traps with public health strategies aimed at a wide range of social determinants of health (and their intersections). Moreover, there is a need to overcome the sense of helplessness that accompanies feelings of being trapped. Research is needed into how to improve Paniyas' capacity to aspire and to benefit from public health interventions [35].

### Next steps

Although the Paniyas were active participants in the PPHA, they have not yet taken advantage of the study findings by assuming a leadership role, such as through advocacy activities, to ensure the findings are used for the community. We are currently working on new strategies to mobilize the Paniyas and increase their capacity to take ownership of the *Paniya Voices *findings. These strategies include providing training in a variety of skills (computers, public speaking, presentations, data interpretation, etc.) for interested Paniyas and identifying public figures from the Paniya tribe who live outside the study site (e.g. political representatives, activists) to speak for and potentially mentor the Paniya community. We hope that, through these strategies, the findings of the study will be heard in the Paniyas' own voices.

## Conclusion

We consider the PPHA to be a potentially useful approach for global health researchers working with marginalized communities to implement research initiatives that will address those communities' health needs in an ethical and culturally appropriate manner. The PPHA can be an alternative approach to assessing health needs that integrates the participants' own perspectives and overcomes perception biases that often arise from health surveys. It can also help to spur marginalized populations to raise their voices beyond the research project itself. Moreover, the information derived from such a participatory approach, which includes indigenous views, experiences and priorities, can be an effective tool for designing appropriate interventions.

## Competing interests

The authors declare that they have no competing interests.

## Authors' contributions

KM conceived and designed the study and undertook the feasibility study. DN and KM developed and directed the field work. HK conducted GIS mapping and oversaw field work. KM, AS, and HK are analyzing the data and working with the participating colonies on data interpretation. SH provided specific input on the theoretical framework and methodological guidance for the study. All authors read and approved the final manuscript.

## Pre-publication history

The pre-publication history for this paper can be accessed here:

http://www.biomedcentral.com/1471-2458/10/149/prepub
